# Point-of-care detection of lactate in cerebrospinal fluid

**DOI:** 10.1186/s40635-021-00385-9

**Published:** 2021-04-06

**Authors:** C. Stephani, A. H. K. Choi, O. Moerer

**Affiliations:** 1 Clinic for Anesthesiology, University Medical Center Goettingen, Robert Koch-Strasse 40, 37075 Goettingen, Germany; 2 Department for Neuroradiology, University Medical Center Goettingen, Robert Koch-Strasse 40, 37075 Goettingen, Germany

**Keywords:** Cerebrospinal fluid, Lactate, Meningitis, Blood gas analyzer, Point-of-care

## Abstract

**Purpose:**

Measurements of cerebrospinal fluid (CSF) lactate can aid in detecting infections of the central nervous system and surrounding structures. Neurosurgical patients with temporary lumbar or ventricular CSF drainage harbor an increased risk for developing infections of the central nervous system, which require immediate therapeutic responses. Since blood gas analyzers enable rapid blood-lactate measurements, we were interested in finding out if we can reliably measure CSF-lactate by this point-of-care technique.

**Methods:**

Neurosurgical patients on our intensive care unit (ICU) with either lumbar or external ventricular drainage due to a variety of reasons were included in this prospective observational study. Standard of care included measurements of leucocyte counts, total protein and lactate measurements in CSF by the neurochemical laboratory of our University Medical Center twice a week. With respect to this study, we additionally performed nearly daily measurements of cerebrospinal fluid by blood gas analyzers to determine the reliability of CSF-lactate measured by blood gas analyzers as compared to the standard measurements with a certified device.

**Results:**

62 patients were included in this study. We performed 514 CSF-lactate measurements with blood gas analyzers and compared 180 of these to the in-house standard CSF-lactate measurements. Both techniques correlated highly significantly (Pearson correlation index 0.94) even though lacking full concordance in a Bland–Altman plotting. Of particular importance, regular measurements enabled immediate detection of central infection in three patients who had developed meningitis during the course of their treatment.

**Conclusion:**

Blood gas analyzers measure CSF-lactate with sufficient reliability and can help in the timely detection of a developing meningitis. In addition to and triggering established CSF diagnostics, CSF-lactate measurements by blood gas analyzers may improve surveillance of patients with CSF drainage.

This study was retrospectively registered on April 20th 2020 in the German trial register. The trial registration number is DRKS00021466.

**Supplementary Information:**

The online version contains supplementary material available at 10.1186/s40635-021-00385-9.

## Introduction

Lactate is an end-product of anaerobic glycolysis. Elevated blood values of lactate often indicate excessive anaerobic cell metabolism, but can also be related to increased glycolysis of neoplastic tissue (Warburg effect), non-neoplastic hyperglycolysis or liver disease. The tight endothelial barrier between blood and cerebrospinal fluid (CSF) largely restricts transfer of blood-lactate into CSF [1]. Accordingly, the primary source of CSF-lactate is endocranial and -spinal. While reference values of CSF-lactate are < 2 mmol/l a CSF-lactate ≥ 4.2 mmol/l is regarded as a strong indicator of a non-viral meningitis [2] with a diagnostic sensitivity and specificity of up to 93–99% and 88–94%, respectively [3, 4]. This has been demonstrated for patients after neurosurgical interventions as well [1, 5, 6] even though there are contradictive results [7, 8]. Within the group of neurosurgical patients, those with a temporary external drainage of the CSF are under particular risk for developing an infection of the CSF and the central nervous system. Therefore, regular CSF analysis is mandatory in this patient group. Unfortunately, CSF analysis requires specialized laboratory facilities, the availability of which often is discontinuous. Therefore, a previous study explored the ability of conventional blood gas analyzers to measure CSF-lactate in patients after a single lumbar puncture and demonstrated a good correlation between values measured by a blood gas analyzer and those measured with an established device in a neurochemical laboratory even though error probability increased with increasing lactate values [9]. Here, we investigated if a conventional blood gas analyzer can reliably measure CSF-lactate in neurosurgical ICU patients with a temporary external CSF drainage.

## Methods

This study was approved by the ethics committee of the University of Göttingen, and conforms to the Declaration of Helsinki. All patients or their legal guardians gave their informed consent to participation in the study.

## Study design

This study was designed as longitudinal observational study. Any patient ≥ 18 years of age with a temporary non-coated lumbar or ventricular catheter and a corresponding drainage (Ventrex@ Neuromedex, Hamburg, Germany) based on an acute neurosurgical indication and being treated on the anesthesiological and surgical intensive care unit of the University Medical Center Göttingen was eligible for inclusion. Inclusion and data collection took place between July 2019 and October 2020. We defined no specific exclusion criteria for this study. The study-related diagnostic measure consisted of nearly daily additional analyses of CSF taken out of the ventricular or lumbar drainage. These regularly began within 24 h after drainage placement. We carefully obtained 400–500 µl of CSF with an uncoated 2-ml plastic syringe under aseptic conditions at the patient-nearest outlet of the drainage. This sample was immediately (i.e., within approximately 2 min) analyzed by a blood gas analyzer (GEM Premier 5000®, Instrumentation Laboratory, Bedford, U.S.A.). In the majority of cases, we repeated the analysis with another blood gas analyzer of the same specification for control purposes immediately after the first analysis. Additionally, and as part of the routine patient care, an arterial blood sample was analyzed using a blood gas analyzer mostly within one hour of analyzing CSF. Independent from that and according to the internal standard operating procedure for CSF analysis in patients with continuous external CSF drainage, we sent a probe of CSF to the neurochemical laboratory of the hospital twice a week. Therefore, for these days we were able to compare CSF analyzed by the internal reference device for CSF diagnostics (respons®910, Diasys, Holzheim, Germany) with that from the blood gas analyzer. If CSF-lactate as determined by blood gas analyzer and on days without regularly planned CSF measurement by the standard method deviated significantly from the reference range or previous measurements, additional CSF was collected and sent for confirmation of the results to our reference laboratory. However, we immediately put pathological CSF results as determined by blood gas analyzer into clinical context and modified treatment if indicated based on all the available data. Likewise, we determined CSF glucose by blood gas analyzers for this study, but did not compare these values to a certified standard method. The given blood gas analyzers measure lactate and glucose amperometrically. An enzymatic reaction of oxygen with metabolites of lactate and glucose, respectively, drives the oxidation of a platinum electrode inducing a current, which thus is proportional to the metabolite concentration. The equation *I* = (*S* * metabolite) + IZ with I being the measured current, *S* standing for the sensitivity and IZ for the reference current, data both of which can be derived from preanalytic settings, therefore allows calculation of the metabolite concentration (information derived from the manufacturers product manual).

### Statistical analysis

To determine the correlations of measured values, we calculated Pearson’s correlation index and compared CSF-lactate as determined by blood gas analysis to CSF-lactate as measured by the internal standard method. Additionally, we compared CSF-lactate and blood-lactate as well as CSF-glucose and blood-glucose each measured by blood gas analyzers in this manner. With respect to determining the accuracy of the measurements, we applied the method of Bland–Altman [10]. For data processing and statistical analysis we used Excel2013® (Microsoft) as well as SPSS26® (IBM).

## Results

**Fig. 1:**
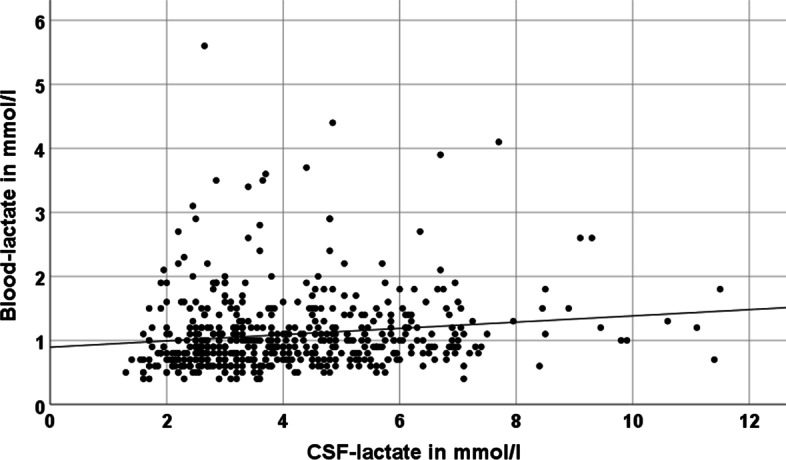
Pearson correlation plot demonstrating the correlation between measurements of lactate in cerebrospinal fluid and blood in mmol/l

**Fig. 2:**
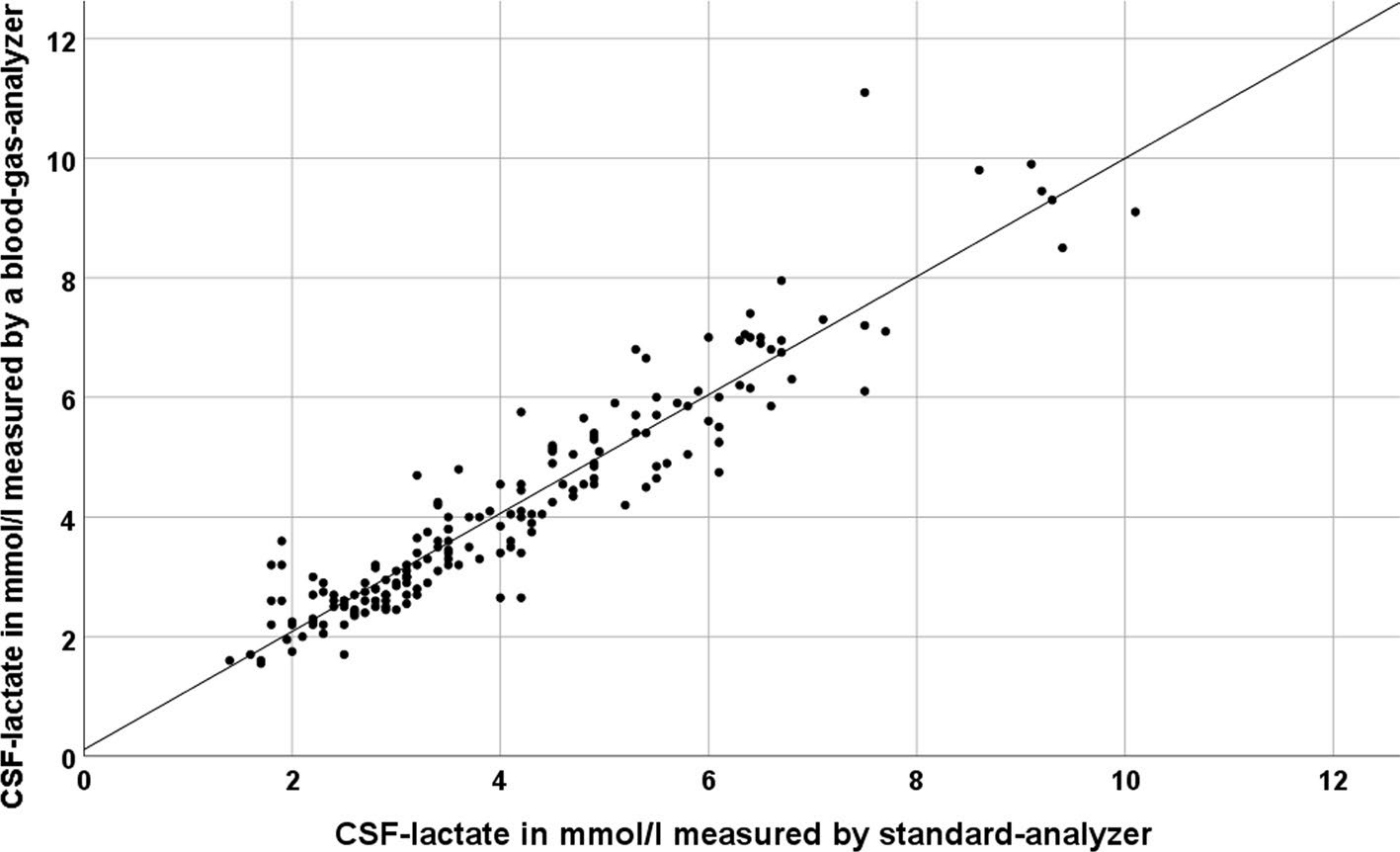
Pearson correlation plot demonstrating the correlation between measurements of lactate in cerebrospinal fluid by the standard method and by the blood gas analyzer in mmol/l

**Fig. 3:**
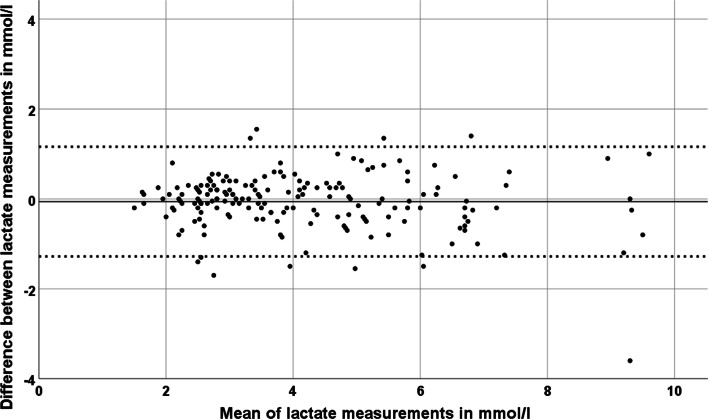
Bland–Altman plot depicting the comparison between cerebrospinal fluid lactate measurements with the standard method and those with the blood gas analyzer by relating the difference between each pair of values to their mean. The dotted lines indicate the range of 1.96 times the standard deviation from the mean of the differences. Aside of 10 marginal, there is only one clear outlier (in the lower right part of the diagram) not within the reference interval of the diagram

We included 62 patients (23 female; mean age 59.5 ± 12.3 years of age) with either lumbar or extraventricular drainage in our study (main diagnoses are given in Table 1). There were 514 complete pairs of CSF-lactate and blood-lactate measurements. In 319 cases (= 62%), we collected CSF from an EVD and in 195 cases (= 38%) from a lumbar drainage. In 292 cases CSF-lactate was measured twice demonstrating high reliability of single CSF-lactate values as measured by blood gas analyzers (Pearson index 0.98). Two pairs of CSF-lactate measurements were eliminated from further analysis due to significant discordance between both lactate and other values indicating a technical or procedural problem. As expected, the correlation between CSF-lactate and blood-lactate was very weak (Pearson index: 0.14), but still statistically significant on a two-sided test-level (*p* = 0.001) (Fig. 1). There were 512 complete pairs of CSF-glucose and blood-glucose measurements. In 289 cases two CSF-glucose measurements were available demonstrating high reliability of single CSF-glucose values as measured by blood gas analyzers (Pearson index 0.98). The Pearson correlation index regarding blood- and CSF-glucose was 0.4 being significant on a two-sided level (*p* < 0.001) (Additional file 1). Descriptive statistics for CSF- and blood-glucose and -lactate are in Table 2. One hundred and eighty sample pairs each with a lactate measurement by a blood gas analyzer and a lactate measurement by the reference method of our neurochemical laboratory were included in the final analysis of feasibility. We excluded one pair of measurements prior to further analysis due to delayed measurement by the reference method. Mean lactate values were 4.13 ± 1.77 mmol/l and 4.19 ± 1.86 mmol/l for reference method and blood gas analyzer, respectively, and thus nearly identical without significant difference (*p* = 0.18). Regarding our main analysis, i.e., comparison between CSF-lactate measured either by the blood gas analyzer or the reference method, we calculated a Pearson correlation index of 0.94 (highly significant correlation on a two-sided significance testing (*p* < 0.001)) (Fig. 2). In order to determine the accuracy or equality of both methods, we prepared a Bland–Altman plot (Fig. 3). Aside of a single outlier, both methods showed a high level of concordance for the full range of measurements with only few differences outside the reference range of 1.96*standard deviation. Three of the 62 patients with continuous external CSF drainage and regular CSF analysis by blood gas analyzer developed a meningitis as diagnosed based on subsequent leucocyte counts in CSF. Based on the higher frequency and availability of testing as compared to our standard procedure, lactate measurements by the blood gas analyzer accelerated making the diagnosis and initiating a therapeutic intervention in these three patients.

**Table 1:** Frequency of main diagnoses of patients with external drainage of cerebrospinal fluid included in this study

Main diagnosis	*N* (female)	age
All diagnoses	62 (23)	59.5 ± 12.3
Subarachnoid hemorrhage	30 (15)	56.6 ± 12.6
Intracranial bleeding	15 (5)	63.8 ± 15.5
Traumatic brain injury	8 (0)	66.9 ± 11.5
Others	9 (3)	54.3 ± 16.1

Other diagnoses were hydrocephalus, subdural bleeding, epidural bleeding, abscess, cerebrospinal fluid fistula, cerebral infarction

**Table 2:** Descriptive statistics of glucose and lactate measurements in cerebrospinal fluid and blood by blood gas analyzers

		CSF	Blood
Glucose (mmol/l) *N* = 512	Mean (standard deviation)	3.7 (± 1.6)	8 (± 2.2)
	Median (min.–max.)	3.8 (0.2–10.5)	7.7 (2.1–19.9)
Lactate (mmol/l) *N* = 514	Mean (standard deviation)	4.1 (± 1.8)	1.1 (± 0.6)
	Median (min.–max.)	3.7 (1.3–11.5)	0.9 (0.4–5.6)

## Discussion

Agreement between lactate measurements by blood gas analyzer and routine CSF measurement was high as indicated by the Pearson correlation index of 0.94. Formally, the resulting Bland–Altman diagram does indicate that the methods compared are not fully interchangeable. However, even though we found no complete agreement between both methods with single values minimally outside the predefined boundaries of agreement (Fig. 3), unlike previously published results [9] our values interestingly demonstrate high agreement for the full range of lactate values, i.e., the correlation did not decrease with increasing lactate values. Hence, this method appears robust as indicator of CSF alterations for the clinically relevant range of CSF-lactate values. Reasons for differences between measurements may include different times between taking CSF and its analysis, which was negligible (around 2 min) in case of measurements by blood gas analyzers, but could take several hours due to internal transport- and processing-times within the hospital with respect to the reference method. Due to its instability at room temperature, prompt measurement of lactate (within 60 min after sampling) is generally preferable. In fact, processing time-related decay may affect CSF-lactate values and indeed, the mean of CSF-lactate measurements by the standard analyzer including the aforementioned delay was lower (4.13 mmol/l) as compared to the results obtained by the blood gas analyzers (4.19 mmol/l) even though this difference was not significant. In order to address this source of possible bias, we should have measured lactate simultaneously by blood gas analyzer and the reference method, which this study did not account for. Obviously, immediacy is a systematic advantage of point-of-care testing. Moreover, the usefulness of timely and frequent lactate measurements as offered by blood gas analyzers becomes particularly apparent in light of those three patients who have had received a drainage for non-infectious maladies of the central nervous system and who later developed a catheter-associated meningitis. Rapid bedside lactate testing detected increases in lactate prior to our reference method, which led to more immediate and accelerated anti-infective treatment as compared to the standard procedure.

Importantly, CSF lactate is an unspecific metabolite and may be elevated in a range of diseases including bacterial and fungal meningitis as well as meningeosis neoplastica [11, 12]. The predominant source of CSF-lactate even in bacterial meningitis is the host organism, i.e., neuronal and immune cells, as studies differentiating d-lactate (prokaryotic) and l-lactate (eukaryotic) in CSF have shown [13] and the positive correlation between leucocyte counts and lactate levels in patients with meningitis supports this finding [14]. Still, a cut-off for CSF-lactate of > 3.5–4.2 mmol/l has demonstrated a high reliability in predicting a non-viral meningitis as confirmed by a recent study [3]. On the other hand, CSF-lactate values alone turned out to be of relatively low predictive value with respect to the development of a postsurgical meningitis in neurosurgical patients [7, 8]. A recent retrospective analysis of 215 CSF-samples of pediatric neurosurgical patients supports this finding. Authors stated especially that the “added value of LCSF for diagnosing CSF infections in children with a history of neurosurgical procedures is unclear and may be influenced by the extent of blood in the CSF” [15]. Indeed, a general limitation of CSF-lactate as a predictor of CSF-infection is the possible contamination of CSF by blood-derived lactate. Almost half of our patients had suffered from subarachnoid hemorrhage often introducing a high amount of blood into CSF. Correspondingly, while blood contamination affects CSF concentrations of amino acids and a group of vitamins only mildly [16] it influences CSF protein diagnostics [17]. Still, in a more experimental setting the addition of different amounts of blood to otherwise normal CSF of 33 adults did not influence the lactate level significantly, but led to higher glucose measurements [18]. In general, single CSF-lactate measurements in post-neurosurgical patients with external CSF drainage, especially in case of major blood contamination, may be less reliable in predicting CSF-infection as compared to otherwise non-contaminated CSF. Nonetheless, by providing a longitudinal view, regular postsurgical measurements of CSF-lactate and -glucose by point-of-care blood gas analyzers may help to rapidly detect inflammatory events in the CSF.

As measurements of blood-glucose by blood gas analyzers is part of the routine for nearly any ICU patient, a pair of CSF- and blood-glucose is easily generated. This is important, since CSF-glucose on its own has a rather poor while a low ratio of CSF-to-blood-glucose of < 0.4 has a well-established predictive value for detecting a non-viral meningitis [19]. Importantly however, and in contrast to the CSF-lactate measurements, we did not control for the accuracy of the blood gas analyzer-derived CSF-glucose measurements by comparing them to results generated by an established CSF-glucose analyzer. Hence, these values so far remain not validated. Still, a recent study demonstrated that point-of-care glucometers can reliably measure CSF-glucose and help in detecting meningitis with a sensitivity of 94% and a specificity of 91% when using a cut-off for the CSF/blood glucose ratio of 0.46 [20]. As outlined before, the major advantage of such measurements by a blood gas analyzer is the immediate availability of results. Additionally though, broad availability of blood gas analyzers would facilitate diagnostic capabilities in resource limited situations as has been previously suggested and is currently under investigation for point-of-care CSF glucometry [21, 22].

## Conclusion

CSF-lactate measurements by a blood gas analyzer may accelerate detection of potential CSF inflammation in patients with temporary external CSF drainage.

## Supplementary Information

**Additional file 1: Fig. S1.** Pearson correlation plot demonstrating the correlation between 512 measurements of glucose in CSF and serum in mmol/l.

## Data Availability

The raw data of the study can be provided upon request.
