# Supplementary material for: Point-of-care detection of lactate in cerebrospinal fluid
Source: Intensive Care Med Exp. 2021 Apr 6;9:18. doi: 10.1186/s40635-021-00385-9 (PMC8024429; doi:10.1186/s40635-021-00385-9)
Supplement: Supplementary file 1 — Additional file 1: Fig. S1. Pearson correlation plot demonstrating the correlation between 512 measurements of glucose in CSF and serum in mmol/l. [file 40635_2021_385_MOESM1_ESM.docx]

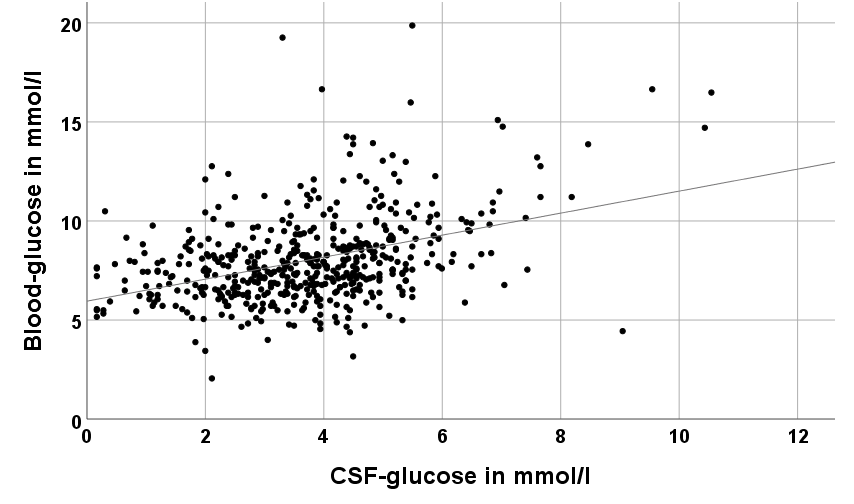


Suppl. 1: Pearson correlation plot demonstrating the correlation between 512 measurements of glucose in CSF and serum in mmol/l.
